# Transvaginal prolapsed submucosal leiomyoma after cesarean section misdiagnosed as bladder prolapse: A case report with literature review

**DOI:** 10.3389/fsurg.2023.1071247

**Published:** 2023-02-13

**Authors:** Ling Li, Jihong Shen, Zhenhua Gao, Xingqi Wang, Daoming Tian, Xunguo Yang, Hang Zhou, Bailuan Li, Dan Tang, Quan Zhang

**Affiliations:** ^1^ The First Department of Urology, The First Affiliated Hospital of Kunming Medical University, Kunming, China; ^2^ Department of Obstetrics, The First Affiliated Hospital of Kunming Medical University, Kunming, China

**Keywords:** submucosal leiomyoma, prolapse, bladder prolapse, postpartum, transvaginal

## Abstract

Uterine leiomyomas, also known as uterine fibroids, are the most common benign tumors found in the female reproductive system. Transvaginal prolapsed submucosal leiomyomas are a rare complication of uterine fibroids during the postpartum period. Due to the lack of sufficient published evidence on these rare complications and their uncommon appearance, they often result in diagnostic and treatment difficulties for clinicians. This case report presents a primigravida with no special prenatal examination developed recurrent high fever and bacteremia following an emergency cesarean section. On the 20th day after delivery, a vaginal prolapsed mass was observed, which was initially misdiagnosed as bladder prolapse before being corrected to a diagnosis of vaginal prolapse of submucosal uterine leiomyoma. This patient was able to retain fertility by prompt use of powerful antibiotics and transvaginal myomectomy rather than undergoing a hysterectomy. For parturient women with hysteromyoma and recurrent fever after delivery where the source of infection cannot be found, the infection of the submucous leiomyoma of the uterus should be highly suspected. It can be helpful to perform an imaging examination to diagnose a disease, and transvaginal myomectomy should be the first choice for treating prolapsed leiomyoma in cases with no obvious blood supply or if pedicle can be achieved.

## Introduction

Leiomyomas, or uterine fibroids, are commonly occurring benign tumors that can be found in the female reproductive system (1). Approximately 10%–30% of pregnant women experience fibroid-related complications (2), such as spontaneous abortion, preterm labor, soft tissue dystocia, uterine inertia, fetopelvic disproportion, fetal malposition, and postpartum hemorrhage (3). Transvaginal prolapsed submucosal leiomyoma, a rare complication among postpartum women, can be difficult for clinicians to diagnose and treat. This case report describes the successful treatment of a postpartum submucosal leiomyoma of the uterus with the vaginal prolapse of a Chinese puerpera (a woman who has recently given birth). It describes the detailed diagnosis and successful treatment process without a hysterectomy.

## Case presentation

**Figure 1:**
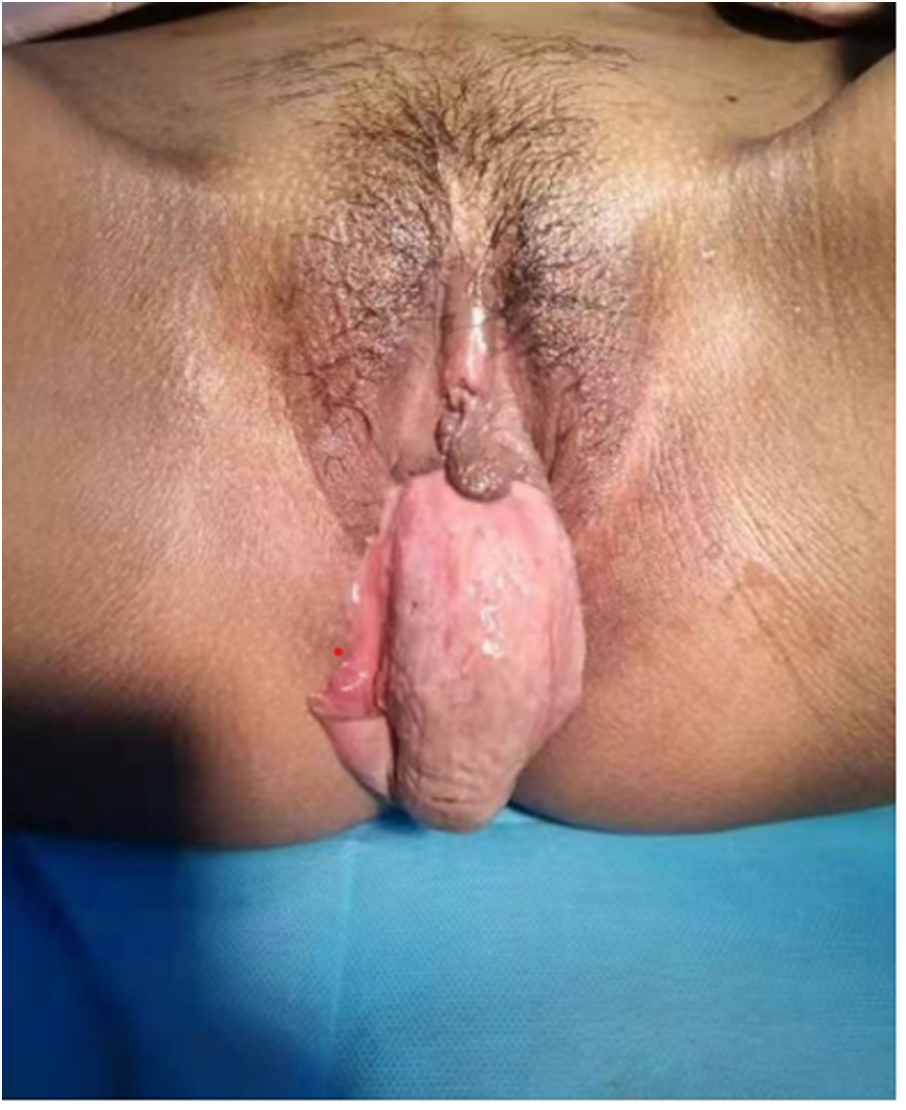
Prolapse day 1. A soft, hyperemia, edema, and bag-like excrescence was observed in the patient’s vulva, which could be self-repaired with a diameter of approximately 4 cm.

The patient, a 24-year-old woman, was referred to our hospital from a subordinate hospital with a persistent, recurrent high fever for 20 days and a prolapsed vaginal mass for 1 day after delivery. At admission, her body temperature was 38.7°C. She had filthy, smelly lochia and a soft, hyperemic, edematous, bag-like excrescence in her vulva (Figure 1), which could be self-repaired and had a diameter of approximately 4 cm.

History: The patient, a gravida 1, para 1, had no special findings during the prenatal examination, including ultrasonic examination. At 40 weeks + 5 days of gestation, she delivered a healthy baby boy weighing 3,300 g *via* emergency cesarean section in a local hospital due to persistent occipital posterior position. While delivering the child, difficulties were encountered in removing the fetal head by hand, resulting in a tear on the left side of the lower segment of the uterus to the external orifice of the cervix. An intramural leiomyoma with a diameter of about 1.5 cm was found around the incision on the anterior wall of the uterus, and a myomectomy was performed. Following the operation, the patient developed a recurrent fever, with the highest temperature of 39.8°C. Her white blood cell count was 21.56 × 10^9^, and the percentage of neutrophils was 85%. Blood culture, urine culture, and vaginal discharge culture all indicated multiple drug resistance to *Escherichia coli*. Based on the drug sensitivity results, it was decided to replace the antibiotics with meropenem 1 g Q8H + metronidazole 0.5 g Q8H + azithromycin 0.5 g QD. During treatment, the patient's peak temperature decreased. At 20 days postpartum, a fleshy tissue emerged from the vagina as the patient was squatting. An ultrasound examination at the local hospital suggested that the tissue may be prolapsed bladder, and the patient was immediately transferred to our hospital. Upon admission, considering the patient's serious infection and the multiple drug resistance to *E. coli*, she was treated with the antibiotic solution of meropenem 1 g Q8H + doxycycline 0.1 g Q12H.

On the second day after the prolapse of the vaginal orifice, the tissue on the surface of the mass had necrotized and exfoliated, accompanied by a stench. To avoid worsening infection caused by ischemic necrosis of the prolapsed “bladder,” the mass was cleaned and disinfected daily before being lubricated with paraffin oil and gently inserted into the vagina. B-ultrasound indicated a strong echo with sound shadow in the intrauterine cavity (indicative of pus formation).

**Figure 2:**
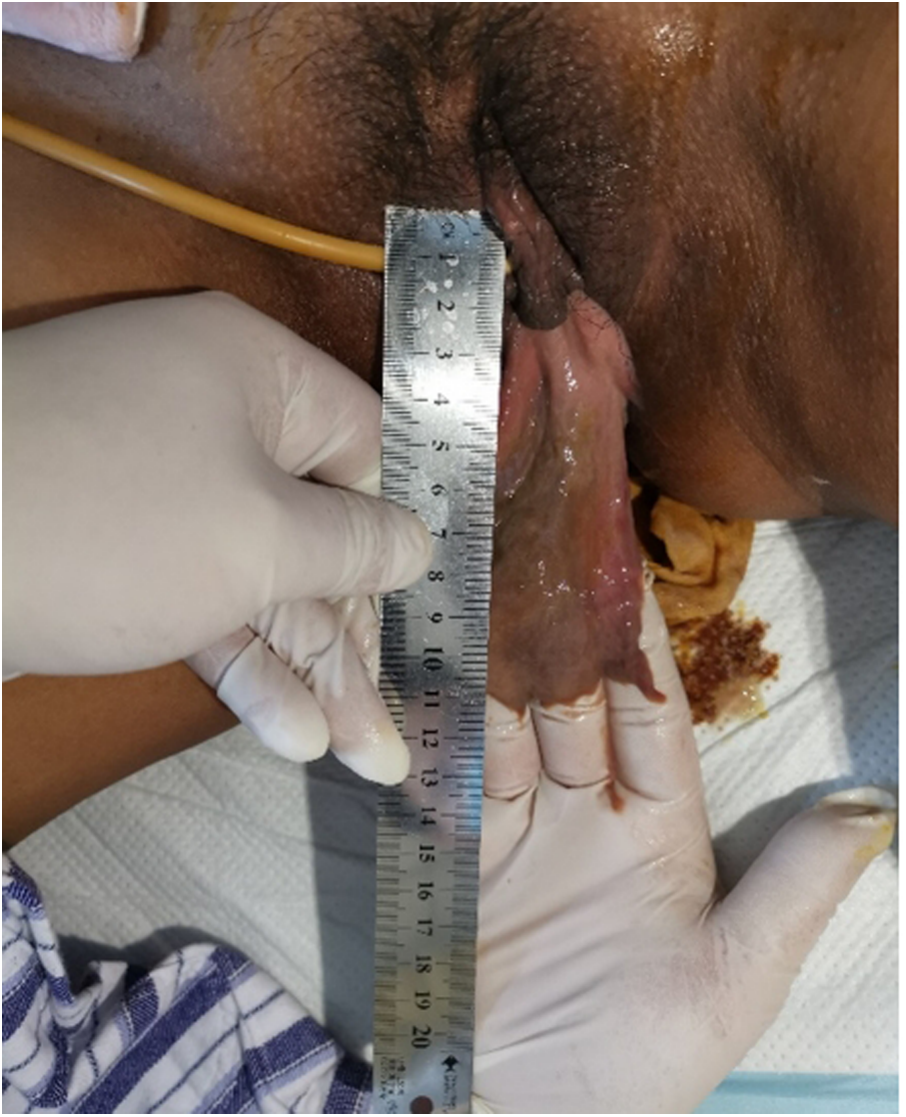
Prolapse day 5. The mass became flaky and gradually darkened due to ischemia.

**Figure 3:**
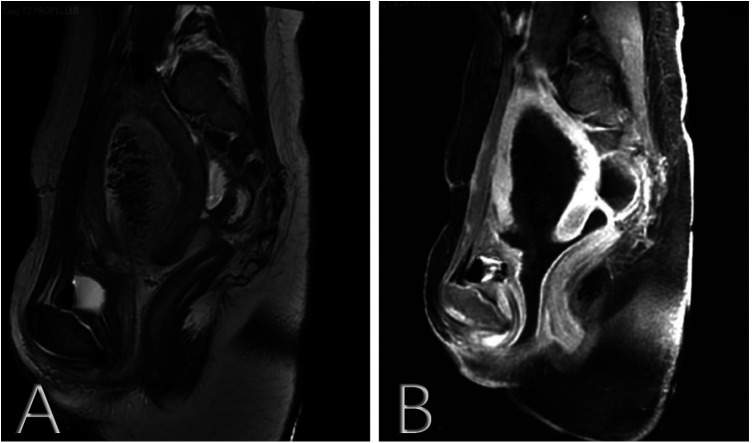
(**A**) T2-weighted MRI images of the pelvis found uneven signals in the myometrium of the uterus, slightly lower signal mass near the bottom of the posterior wall of the uterus, no diffusion limitation in DWI, slightly uneven lower signal in the intrauterine, scattered gas signal, and slightly uneven lower signal in the vagina connected with the intrauterine signal. (**B**) Dynamically enhanced sagittal T1-weighted images of the pelvis found no enhancement of the intrauterine and vaginal signals. MRI, magnetic resonance imaging; DWI, diffusion-weighted imaging.

On the fifth day after the prolapse, the mass became flaky and gradually darkened due to ischemia (Figure 2). The validity of the bladder prolapse diagnosis was questioned, requiring a multidisciplinary consultation given the patient's absence of dysuria and urine retention. T2-weighted magnetic resonance imaging (MRI) images of the pelvis showed uneven signals in the myometrium of the uterus, moderately lower signal mass near the bottom of the posterior wall of the uterus, no diffusion limitation in diffusion-weighted imaging (DWI), marginally unbalanced lower signal in the intrauterine, scattered gas signal, and slightly uneven lower signal in the vagina connected with the intrauterine signal (Figure 3A). Dynamically enhanced sagittal T1-weighted images of the pelvis showed no enhancement of the intrauterine and vaginal signal (Figure 3B). At this stage, bladder prolapse had been ruled out, and the patient was first diagnosed with a prolapsed submucosal fibroid. Once the diagnosis was confirmed, transvaginal resection of the protuberant was performed with the assistance of an obstetrician.

**Figure 4:**
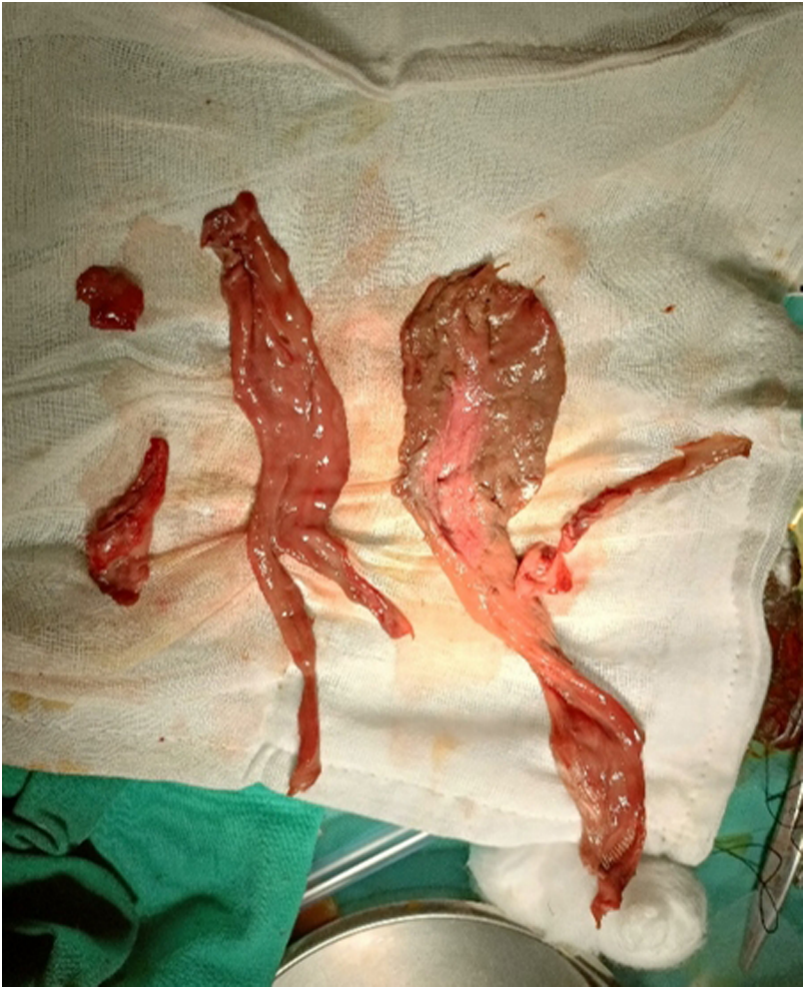
Complete excision of the vegetation was removed as far as possible and divided into several pieces of irregular tissue during the operation.

Operation process: The patient had a 16F urethral catheter removed under general anesthesia. Upon examination, the uterus was observed to be intact and large, consistent with the size of the uterus 30 days after delivery. Exploration revealed an irregular, prolapsed vegetation, approximately 18 cm long, 10 cm wide, and 4 cm thick, originating from the uterine cavity. The cervical edge could be touched with careful palpation. The irregular vegetation was gripped and pulled hard by oval pliers using vaginal retractors to expose the field of vision, and the pedicle of the vegetation was found to be deep and strong. A transabdominal ultrasound showed the vegetation to be connected to the fundus uteri. The vegetation was excised completely as far as possible and divided into irregular tissue pieces during the operation (Figure 4). An old laceration up to the vaginal fornix on the right part of the cervix was sutured using 2-0 absorbable sutures.

Histopathologic examination found a braided arrangement of muscular tissue with complete coagulation necrosis, which confirmed the diagnosis of leiomyoma with coagulation necrosis.

## Literature review

The PubMed database was searched using MeSH terms including “submucosal fibroids,” “submucosal leiomyoma,” “submucous leiomyoma,” “submucous myoma,” “postpartum,” “after delivery,” or “caesarean” from January 1945 to November 2021. Each article abstract was evaluated, and the relevant original English language articles were read. Relevant articles were cross-referenced to identify related studies or publications on the subject, and cases of transvaginal prolapse of uterine submucous myoma or cervical myoma after delivery since 1949 were summarized (1, 3–13) (Table 1). The literature review found that transvaginal prolapse of postpartum submucosal fibroids occurred from immediate postpartum to seven weeks postpartum, with 7 of the 14 cases occurring at 4–5 weeks postpartum. Regarding symptoms, 12 of the 14 cases had a postpartum fever or spasmodic abdominal pain. Additionally, some patients experienced vaginal bleeding and malodorous vaginal secretions, and a few experienced urinary retention and urethrovaginal fistulas. The prolapsed fibroids were mostly pedicled, soft, and irregular. The location of their pedicles was found to be distributed in all uterine walls, but most were around the cervical canal and fundus. Transvaginal myomectomy was the most common surgical method, but some patients had to undergo transvaginal/transabdominal hysterectomy as a result of incomplete resections or serious complications. In both patients and infants, postoperative outcomes were positive, with no deaths reported.

**Table 1:** Summary of the cases with transvaginal prolapse of uterine submucous myoma or cervical myoma after delivery since 1949.

Author (publication year)	Clinical manifestations	Fibroid description	Location	Time after delivery (delivery mode)	Management	Patient and fetal outcome	Pathology
Gainey and Keeler (7) (1949)	Abdominal cramps	An oval-shaped tumor mass, size 5 cm × 3 cm	…	5 h (natural labor)	Spontaneous expulsion	Normal postoperative course, healthy infant	Rapidly proliferating edematous fibromyoma with early necrosis and degeneration
Anderson and Lauderdale (6) (1964)	Fever (up to 39.4°C), severe cramps, vaginal bleeding, and becoming conscious of a mass in the vagina	Mass was soft in consistency and foul-smelling	In the uterine cavity	4 weeks (natural labor)	Blunt and sharp dissection	Normal postoperative course and has subsequently been delivered without complications, healthy infant	Degenerating leiomyoma
	Fever (up to 40.0°C), vaginal bleeding	…	Above the internal os of the cervix and involved the left lateral and anterior uterine walls	25 days (natural labor)	Supracervical hysterectomy and left salpingo-oophorectomy	Normal postoperative course, healthy infant	Endocervix showing an inflammatory reaction; leiomyoma, intramural, with secondary degeneration; and subacute endometritis and metritis
	Fever (up to 38.9°C), abdominal pains, vaginal bleeding, and a mass protruding from the vagina	A necrotic, foul-smelling myoma, size 10 cm × 12 cm	On the anterior to the vertex	10 days (natural labor)	Vaginal myomectomy	Normal postoperative course, menses occurred regularly, healthy infant	Leiomyoma with necrosis and focal hemorrhage
Mason (3) (2002)	Urinary retention, vaginal bleeding with cramping	A large necrotic mass protruding through the os extending to the introitus, size 15 cm × 15 cm × 3 cm	Attached to the anterior lower uterine segment by a 4-cm stalk	4 weeks (natural labor)	Blunt and sharp dissection	Normal postoperative course, healthy infant	Degenerating leiomyomata
Thorpe-Beeston and Sebire (8) (2002)	…	Smooth and firm, measuring 8 cm in diameter	…	After delivery (preterm labor)	Spontaneously delivered	Normal postoperative course, healthy infant	Benign leiomyoma
Murakami et al. (10) (2007)	Fever, genital bleeding	Part of the cervical myoma had been sloughed off into the vagina, weight 1,140 g	On the lower posterior wall of the cervix (early pregnancy)	16 days (cesarean), the myoma threatened to obstruct vaginal delivery	It was resected twice, followed by abdominal myomectomy	Normal postoperative course, healthy infant	Degenerating leiomyoma
Pieh-Holder et al. (12) (2014)	Persistent vaginal bleeding	A 5-cm prolapsed vaginal fibroid with uterine inversion	…	7 weeks (natural labor)	Transvaginal hysterectomy	Normal postoperative course, healthy infant	Degenerating endometrium with stromal bleeding and intramural leiomyoma with severe ischemic changes
DeMaio and Doyle (4) (2015)	Fever (up to 38.9°C), a feeling of something coming down	A large, exceedingly malodourous mass, size 27 cm × 10 cm	In the uterine cavity	5 weeks (natural labor)	Manually removed	Normal postoperative course, menses occurred regularly, healthy infant	Degenerating leiomyomata
Sagoo et al. (9) (2015)	Fever, abdominal cramps, and pain with foul smelly vaginal discharge	A large fleshy vaginal mass was protruding from the patient’s vagina	The pedicle was hanging from the uterine fundus	5 weeks (emergency cesarean)	Was twisted off per vaginal route	Infant with multiple congenital abnormalities and severe intrauterine growth restriction	…
Elgonaid et al. (5) (2017)	Severe lower abdominal pain and foul smelly vaginal secretions	A smelly large fleshy mass with a friable surface bulging from the cervix but not protruding into the vagina	In the uterine cavity and attached to the fundus	35 days (natural labor)	Manual removal per vaginal route	Healthy infant	Infarcted leiomyoma
Zhang et al. (1) (2018)	Fever (up to 39.2 °C), a mass extrusion in the vaginal orifice, and smelly discharge	An irregular, long, thin, and broad sarcoid mass hung down through the vaginal orifice, size 38.0 cm × 6.0 cm × 2.0 cm	On the upper right side of the cervical canal, near the internal ostium	12 days (cesarean), lying transverse to the fetus	Blunt and sharp dissection	Has subsequently been delivered without complication, healthy infant	Degenerating leiomyoma with infarction and no atypical features
Nkwabong (13) (2018)	Fever (up to 38.5 °C), hypogastric tenderness with foul-smelling vaginal discharge, and urinary retention	A mass of approximately 10 cm in diameter was present in the vagina	On the posterior uterine wall at 3 cm from the external cervical os	5 weeks (natural labor)	Was twisted off per vaginal route	Developed a urethrovaginal fistula, healthy infant	Uterine leiomyoma
Wang et al. (11) (2021)	Spontaneous miscarriage, fever (up to 39.6 °C), and lower abdominal pain	The fibroids had degenerated and necrosed, and a large heterogeneous echoic pattern bulged into the vagina, size 17 cm × 10 cm × 9 cm	In the uterine cavity	40 days after a miscarriage	Transvaginal mass resection, followed by transfer to exploratory laparotomy	Has subsequently been delivered without complications	A fibroid that demonstrated significant degeneration and suppurative necrosis

## Discussion

It is estimated that uterine leiomyomas occur in 20%–40% of women during their reproductive years (14). During pregnancy, women with uterine fibroids have a higher risk of preterm delivery, placenta previa, postpartum hemorrhage, and cesarean section compared to those without uterine fibroids (15). Furthermore, due to the rarity of the complication of myoma transvaginal prolapse, diagnostic difficulties or misdiagnosis can occur if an insufficient auxiliary examination is performed. In this case, large uterine fibroids were not discovered by B-ultrasonography during regular labor examination, which could be related to edema and the softening of fibroids.

There are four major reasons for the initial misdiagnosis of bladder prolapse in this case. First, the clinical manifestations of the patient—the prolapsed mass being slightly smooth and pouch-like—make it difficult to distinguish from a prolapsed bladder based on appearance. Second, this disease is rare and clinicians had not previously encountered similar cases, resulting in misdiagnosis. Third, as the ultrasound examination of the patient during pregnancy did not reveal the presence of uterine fibroids, the sonographer at the subordinate hospital offered a misdiagnosis of bladder prolapse. Fourth, due to the serious condition of the patient upon admission, the relevant examination was not completed in a timely manner.

The specific mechanism of uterine leiomyoma prolapse is unclear; however, according to the case analysis, factors such as treatment with uterine artery embolization (UAE), laparoscopic-assisted uterine depletion (LAUD), and gonadotropin-releasing hormone adjuvant therapy (GnRH-a) may play a role (10). Additionally, following delivery, the uterus returns to its pre-pregnancy size within approximately 4 weeks. Strong postpartum uterine contractions are also believed to be a factor that promotes fibroid discharge (10). In addition, Demirci et al. (16) discovered that when submucosal myomas have a pedicle in the uterine cavity, the natural tendency for the uterus is to attempt to expel the soft myomas through the cervical canal, ultimately leading to cervix dilation. Myomas are generally necrotic and can sometimes become infected when emerging from the gradually expanding cervix since they have difficulty maintaining an adequate blood supply through the long pedicle (5). In this case, it is possible for a quite large submucosal pedunculated myoma to be delivered gradually through a visibly dilated cervix, resulting in gradual ischemic necrosis.

Clinical diagnosis of vaginal prolapse of the submucosal myoma can be challenging due to its occult nature and rarity. However, radiology can help make a diagnosis. Computed tomography (CT) scans can reveal a qualitative echo of a pelvic mass, linking solid and cystic components to degeneration and infarcted areas, which can lead to an early and definite diagnosis (11). MRI can provide significantly accurate and useful information related to myoma survival, vascular supply, and myometrial involvement and also plays an important role in selecting the appropriate surgical procedure (17). In this case, an enhanced MRI revealed that there was no blood supply to the prolapsed fibroid and its pedicle, and thus, a transvaginal myomectomy was chosen.

There are currently no clinical guidelines for treating a pedunculated submucosal myoma during pregnancy or postpartum, as this type of myoma is rare, and there have only been 14 cases reported since 1945, all of which have been accompanied by an occult development of suppuration (1–13). Sirha et al. (18) have found that surgical intervention combined with more effective antibiotics is the most appropriate treatment. Golan et al. (19) found that the success rate of vaginal myomectomy was 95.6% in 46 patients with prolapsed submucosal myomas over a 10-year period and the recurrence rate was incredibly low. Therefore, it is recommended that transvaginal myomectomy should be the first choice of surgical treatment. Surgically preserving fertility may be possible if the surgeon reaches the uterine pedicle (16).

In summary, the infection of submucous leiomyoma of the uterus should be highly suspected in parturient women with hysteromyoma and recurrent fever after delivery, where the source of infection cannot be identified. MRI can provide incredibly accurate and useful information related to myoma survival, vascular supply, and myometrial involvement and plays a crucial role in selecting surgical procedures. Transvaginal myomectomy may be the preferred treatment for prolapsed myoma in situations where the pedicle can be achieved.

## Data Availability

The original contributions presented in the study are included in the article/Supplementary Material, further inquiries can be directed to the corresponding author.
